# The Strong Correlation Between Multiple Births and Preterm Birth Rates in Greece From 1991 to 2022

**DOI:** 10.7759/cureus.68983

**Published:** 2024-09-09

**Authors:** Nikolaos Vlachadis, Dionysios N Vrachnis, Nikolaos Loukas, Nikolaos Antonakopoulos, Panagiotis Peitsidis, Marios Mamalis, Panagiotis Antsaklis, Marianna Theodora, George Daskalakis, Nikolaos Vrachnis

**Affiliations:** 1 Department of Obstetrics and Gynecology, General Hospital of Messinia, Kalamata, GRC; 2 Third Department of Obstetrics and Gynecology, National and Kapodistrian University of Athens, Attikon Hospital, Athens, GRC; 3 Department of Obstetrics and Gynecology, Tzaneio Hospital, Piraeus, GRC; 4 Department of Obstetrics and Gynecology, School of Health Sciences, University of Patras, Patras, GRC; 5 Fifth Department of Obstetrics and Gynecology, Elena Venizelou Maternity Hospital, Athens, GRC; 6 First Department of Obstetrics and Gynecology, National and Kapodistrian University of Athens, Alexandra Hospital, Athens, GRC

**Keywords:** greece, multiple births, national trend, preterm birth, preterm birth rate

## Abstract

Background

This study aims to investigate the correlation between the rising preterm birth rate (PBR) in Greece from 1991 to 2022 and the incidence of multiple births.

Methodology

Official data on live births in Greece from 1991 to 2022 were sourced from the Hellenic Statistical Authority. The PBR, defined as the number of live births occurring at <37 gestational weeks, and the multiple birth rate (MBR), representing live births from multifetal gestations, were calculated per 100 total live births. The relationship between the PBR and the MBR was evaluated using the non-parametric Spearman’s rank correlation coefficient (rho). This association was confirmed through linear regression models, with MBR as the independent variable and PBR as the dependent variable, calculating the beta coefficient (β) and the coefficient of determination (R-squared).

Results

A very strong direct positive correlation was identified between PBR and MBR throughout the study period, with a Spearman’s rho of 0.950 (p < 0.001). This conclusion was further supported by the linear regression model, which yielded a β coefficient of 3.32 (95% confidence interval = 2.78 to 3.86, p < 0.001). The R-squared was 0.838, indicating that the change in MBR explained 83.8% of the rise in PBR. The strongest correlations were observed for moderate PBR (32-33 weeks) with a rho of 0.962 (p < 0.001) and late PBR (34-36 weeks) with a rho of 0.940 (p < 0.001). During the period of a steep increase in prematurity rates in the country (1991-2011), an almost perfect correlation between PBR and MBR (rho = 0.987, p < 0.001) was noted. However, in recent years (2011-2022), characterized by a marginal increase in PBR, this association diminished, with a rho of 0.655 (p = 0.021).

Conclusions

This analysis revealed a strong positive correlation between the PBR and MBR in Greece from 1991 to 2022, underscoring the significant impact of multiple pregnancies on the substantial increase in preterm births within the Greek population.

## Introduction

Preterm labor, occurring before 37 weeks of gestation, represents a significant pregnancy complication with severe implications for both the newborn and the mother. These consequences range from elevated mortality in the early days of life to long-term morbidity that may persist into adulthood. Furthermore, a prior history of preterm birth is a recognized risk factor for metabolic and cardiovascular issues in the mother. Collectively, these sequelae contribute to substantial health and economic burdens, positioning preterm birth as a critical public health concern and a significant challenge for perinatal medicine [1-3].

Preterm labor can occur spontaneously or be provider-initiated through induction of labor or elective cesarean section for medical reasons [4]. Preterm labor constitutes a complication of pregnancy that is considered to be of multifactorial etiology, with intrauterine infection being a substantial factor [5,6]. Recent studies have focused on the determination of potential risk factors contributing to preterm delivery [7]. Globally, one out of every 10 babies is born prematurely, with preterm birth rates (PBRs) showing minimal improvement over the past decade and, in certain regions, even increasing. It is estimated that close to 1 million newborns died in 2021 from complications of prematurity. Notably, there has been no significant change in global PBRs over the past decade, and although a small number of countries have managed to slightly decrease their prematurity rates, the overall impact remains limited [1,8,9]. In 2010, approximately 15 million children were born prematurely worldwide, accounting for around 11% of all births. This trend showed an increase over the preceding two decades, with significant disparities observed between affluent and impoverished nations [8]. By 2020, the global proportion of preterm births decreased to 10%, equating to an estimated over 13 million newborns born prematurely [9].

Epidemiological evidence indicates that within developed nations, variations in prematurity indicators primarily stem from clinical practices to indicate preterm births and fertility treatments, resulting in a rise in the occurrence of multiple pregnancies [10,11]. The escalation of multifetal gestations in recent years has significantly influenced the rise of prematurity rates, given the substantially elevated risk of preterm delivery associated with these pregnancies [12,13]. Several factors contribute to an elevated risk of preterm birth in multiple pregnancies, often working in conjunction. Pathological mechanisms encompass increased uterine and cervical distension, potentially leading to premature contractions, placental insufficiency, and a higher incidence of maternal and fetal complications, such as pre-eclampsia, gestational diabetes, and fetal growth restriction [12-14].

Greece has suffered an epidemic rise in premature births since 1991, reaching rates higher than all other advanced countries. The upward trend was rapid in the period 1991-2011 and plateaued during 2011-2022 [2]. The vast increase in prematurity rates in Greece coincided with a substantial increase in the rates of multiple births, reaching the highest levels globally [15]. This study seeks to investigate the relationship between the escalating trend of preterm birth and multiple birth rates (MBRs) to evaluate the effect of plural pregnancies on the exacerbation of prematurity in Greece.

## Materials and methods

Official data on live births in Greece, categorized by gestational age in completed weeks, and separate data on births by multiplicity were acquired for the years 1991 to 2022 from the Hellenic Statistical Authority [16]. The PBR, denoting live births occurring before 37 weeks of gestation, and the MBR, representing live births from multifetal gestations, were calculated per 100 total live births.

Moreover, PBRs were segmented into the following four subcategories according to gestational age: the extremely preterm birth rate (EPBR) for births before 28 weeks, the very preterm birth rate (VPBR) for births at 28 to 31 weeks, the moderately preterm birth rate (MPBR) for births at 32 to 33 weeks, and the late preterm birth rate (LPBR) for births at 34 to 36 weeks gestation.

The data were analyzed using SPSS Statistics version 22 (IBM Corp., Armonk, NY, USA) and Microsoft Excel version 2010 (Microsoft Corp., Redmond, WA, USA) software. The findings are presented as medians with interquartile range (IQR) (25th and 75th percentiles) and percentages. Year-to-year absolute and relative (%) changes in PBR and MBR were calculated, comparing each year with the preceding one. The correlation analysis between PBR and MBR was conducted for the entire period from 1991 to 2022, as well as separately for the periods 1991-2011 and 2011-2022. A non-parametric Spearman’s rank correlation coefficient (rho) was employed to evaluate the relationship between PBR and the MBR, as the data were non-normally distributed. Furthermore, linear regression models were utilized to investigate the association between MBR (independent variable) and PBR (dependent variable) in greater depth and to quantify the strength of the association. The beta coefficient (β) and the coefficient of determination (R-squared, R^2^) were calculated, along with 95% confidence intervals (CIs). A two-tailed statistical significance level was set at p-values <0.05.

## Results

**Figure 1:**
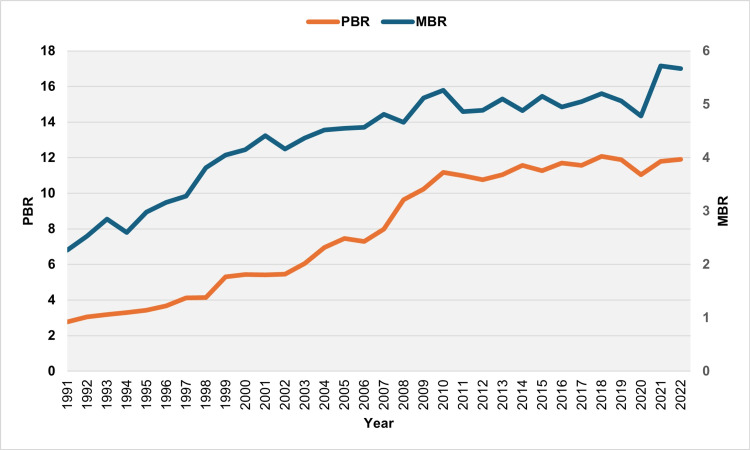
Preterm birth and multiple birth rates (per 100 live births) in Greece, 1991-2022. PBR: preterm birth rate; MBR: multiple birth rate

The PBR in Greece saw a significant rise from 2.77 per 100 live births in 1991 to a peak of 12.07 per 100 live births in 2018, before slightly decreasing to 11.90 per 100 live births in 2022 (Figure 1).

**Figure 2:**
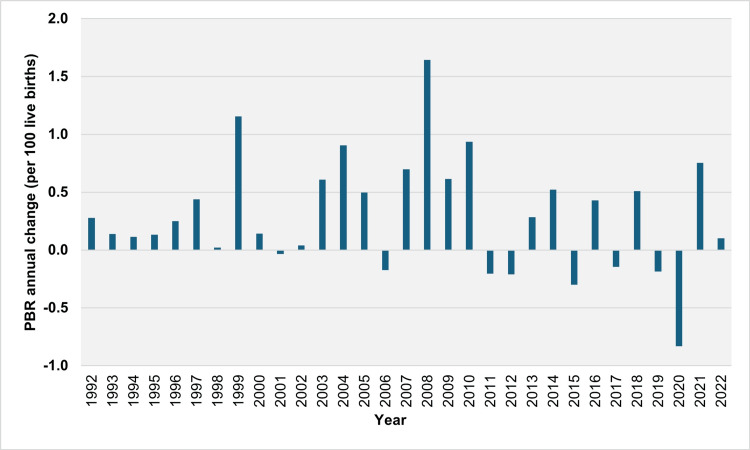
Year-to-year absolute change in the preterm birth rate (per 100 live births) in Greece, 1992-2022. PBR: preterm birth rate

**Figure 3:**
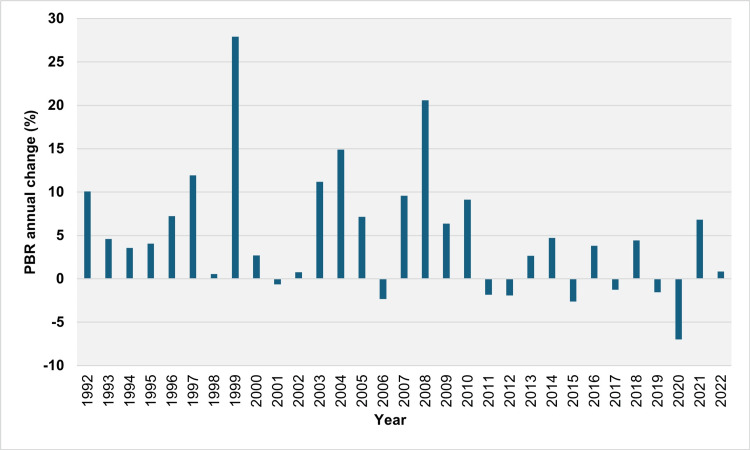
Year-to-year relative (%) change in the preterm birth rate in Greece, 1992-2022. PBR: preterm birth rate

Over 30 years (1992-2022), the PBR increased over 23 years and decreased over eight years (2001, 2006, 2011, 2012, 2015, 2017, 2019, and 2020). The median absolute annual increase in PBR was 0.44 per 100 live births (IQR = 0.14 to 0.70 per 100 live births). The largest absolute increases occurred in 2008, 1999, and 2010, with values of 1.64, 1.16, and 0.94 per 100 live births, respectively. The median relative annual increase in the PBR was 6.4% (IQR = 3.6% to 10.1%), with the most significant relative annual increases observed in 1999, 2008, and 2004 at 27.9%, 20.6%, and 14.9%, respectively. The PBR showed a consistent increase over nine consecutive years, from 1992 to 2000, nearly doubling from 2.77 per 100 live births in 1991 to 5.44 per 100 live births in 2000. Overall, the PBR increased in 17 out of 19 years from 1992 to 2010. The largest absolute annual declines in PBR were recorded in 2020, 2015, and 2012, with values of 0.83, 0.30, and 0.21 per 100 live births, respectively. The most significant relative annual declines were observed in 2020, 2015, and 2006 at 7.0%, 2.6%, and 2.3%, respectively (Figures 2, 3).

**Figure 4:**
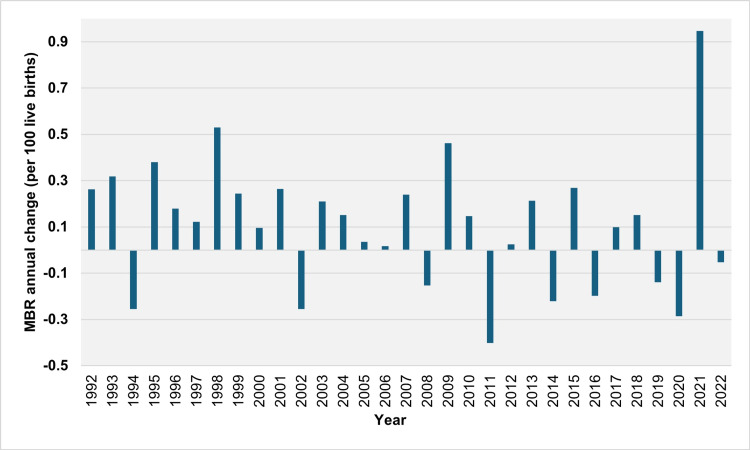
Year-to-year absolute change in the multiple birth rate (per 100 live births) in Greece, 1992-2022. MBR: multiple birth rate

**Figure 5:**
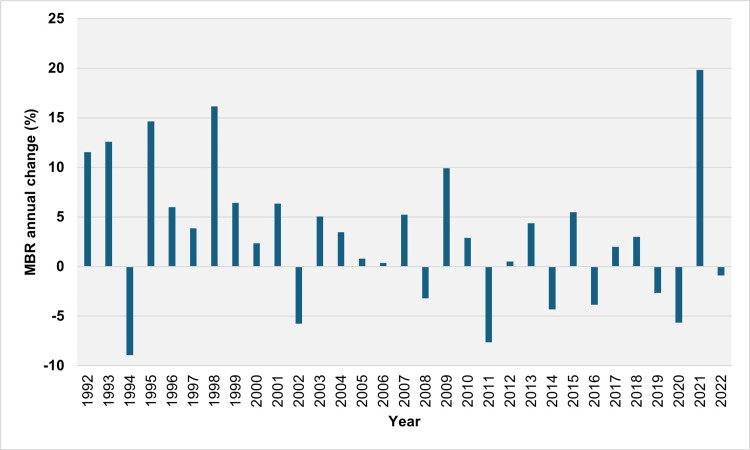
Year-to-year relative (%) change in the multiple birth rate in Greece, 1992-2022. MBR: multiple birth rate

The MBR stood at 2.27 per 100 live births in 1991, reaching a peak of 5.72 per 100 live births in 2021 before slightly decreasing to 5.67 per 100 live births in 2022 (Figure 1). From 1992 to 2022, the MBR increased over 22 years and decreased over nine years (1994, 2002, 2008, 2011, 2014, 2016, 2019, 2020, and 2022). The median annual absolute increase in MBR was 0.21 per 100 live births (IQR = 0.12 to 0.28 per 100 live births). The largest absolute annual increases were observed in 2021, 1998, and 2009, with values of 0.95, 0.53, and 0.46 per 100 live births, respectively. The median annual relative increase in MBR was 5.1% (IQR = 2.7% to 10.3%), with the most significant relative annual increases occurring in 2021, 1998, and 1995 at 19.8%, 16.2%, and 14.6%, respectively. The MBR consistently increased over seven consecutive years, from 1995 to 2001, resulting in a total rise of 69.9% from 2.60 per 100 live births in 1994 to 4.41 per 100 live births in 2001. The MBR increased in 16 out of 19 years from 1992 to 2010. Conversely, the largest absolute annual declines in MBR were observed in 2011, 2020, and 2002, with values of 0.40, 0.29, and 0.26 per 100 live births, respectively, while the most significant relative annual declines were recorded in 1994, 2011, and 2002 at 8.9%, 7.6%, and 5.7%, respectively (Figures 4, 5).

**Figure 6:**
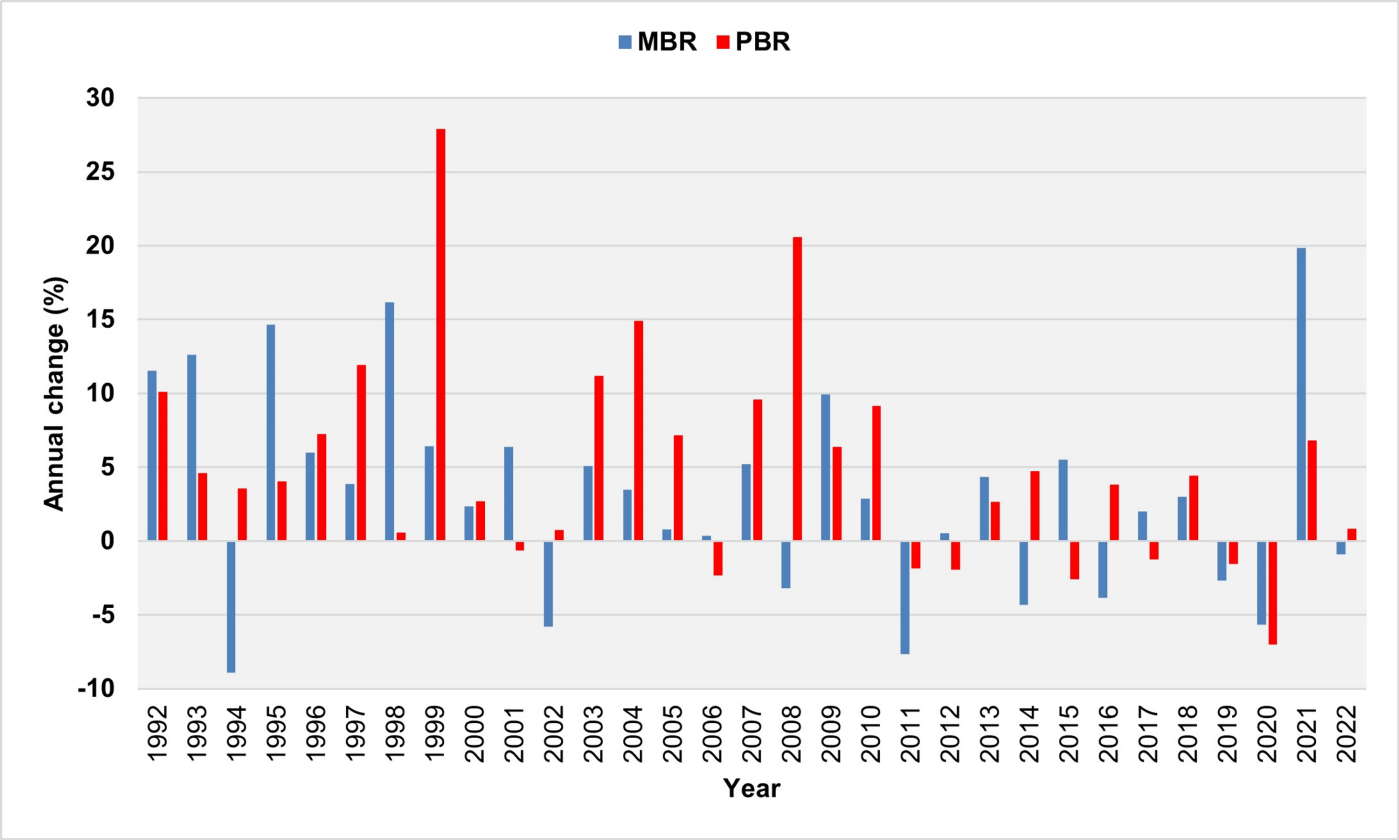
Year-to-year change relative (%) change in PBR and MBR in Greece, 1992-2022. PBR: preterm birth rate; MBR: multiple birth rate

The analysis of the annual changes in PBR and MBR from 1992 to 2022 revealed that in 17 out of 23 years, the rise in PBR was accompanied by a corresponding increase in MBR (Figure 6).

**Figure 7:**
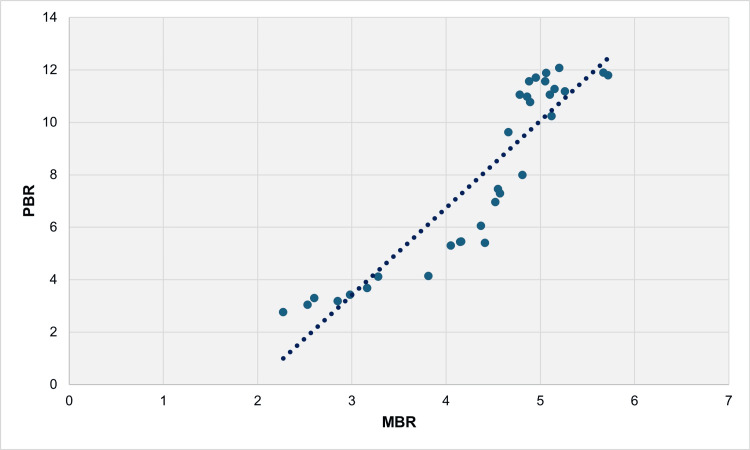
Scatter plot for PBR and MBR (per 100 live births) with the best fit regression line, 1991-2022. PBR: preterm birth rate; MBR: multiple birth rate

From 1991 to 2022, there was a very strong direct positive correlation between PBR and MBR, indicated by Spearman’s rho of 0.950 (p < 0.001). In the linear regression model, the β coefficient was calculated as 3.32 (95% CI = 2.78 to 3.86, p < 0.001), signifying that an increase in MBR by one multiple live births per 100 live births led to an increase in PBR by 3.32 preterm live births per 100 live births. The R^2^ coefficient was determined to be 0.838, indicating that 83.8% of the variability of PBR could be explained by changes in MBR (Figure 7, Table 1).

**Table 1:** Correlation of preterm birth rates with the multiple birth rate in Greece, 1991-2022. PBR: preterm birth rate; EPBR: extremely preterm birth rate; VPBR: very preterm birth rate; MPBR: moderately preterm birth rate; LPBR: late preterm birth rate

Rate	Spearman’s rho	P-value	β	95% confidence interval	P-value	R^2^
PBR	0.950	<0.001	3.32	2.78 to 3.86	<0.001	0.838
EPBR	0.847	<0.001	0.05	0.04 to 0.07	<0.001	0.725
VPBR	0.810	<0.001	0.10	0.07 to 0.13	<0.001	0.570
MPBR	0.962	<0.001	0.37	0.31 to 0.43	<0.001	0.858
LPBR	0.940	<0.001	2.80	2.34 to 3.26	<0.001	0.837

Correlations with MBR were statistically significant across all PBRs, with particularly strong associations observed for MPBR (rho = 0.962, p < 0.001) and LPBR (rho = 0.940, p < 0.001), and slightly weaker correlations for EPBR (rho = 0.847, p < 0.001) and VPBR (rho = 0.810, p < 0.001). In the linear regression models, the β coefficients were calculated as 0.05 (95% CI = 0.04 to 0.07, p < 0.001) for EPBR, 0.10 (95% CI = 0.07 to 0.13, p < 0.001) for VPBR, 0.37 (95% CI = 0.31 to 0.43, p < 0.001) for MPBR, and 2.80 (95% CI = 2.34 to 3.26, p < 0.001) for LPBR. The variations in MBR accounted for 85.8%, 83.7%, and 72.5% of the increases in MPBR, LPBR, and EPBR, respectively, while only 57.0% of the rise in VPBR was attributed to MBR fluctuations (Table 1).

**Figure 8:**
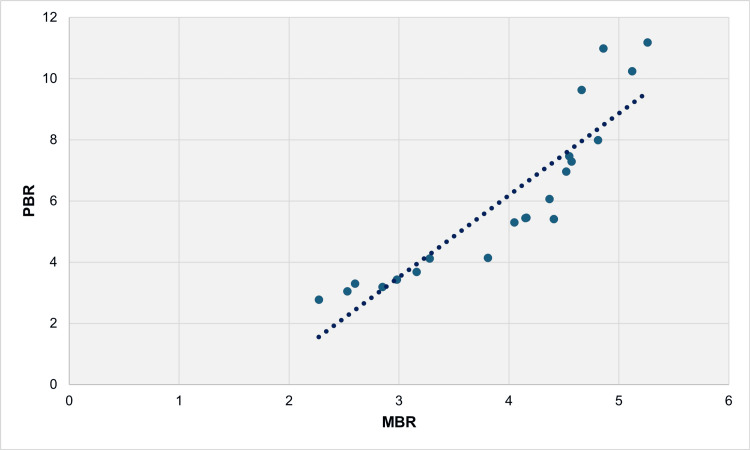
Scatter plot for PBR and MBR (per 100 live births) with the best fit regression line, 1991-2011. PBR preterm birth rate; MBR multiple birth rate

During the period 1991-2011, there was an almost perfect association between PBR and MBR, indicated by a rho of 0.987 (p < 0.001). The linear regression model revealed a β coefficient of 2.68 (95% CI = 2.06 to 3.29, p < 0.001) with an R^2^ value of 0.813 (Figure 8, Table 2).

**Table 2:** Correlation of preterm birth rates with the multiple birth rate in Greece, 1991-2011. PBR: preterm birth rate; EPBR: extremely preterm birth rate; VPBR: very preterm birth rate; MPBR: moderately preterm birth rate; LPBR: late preterm birth rate

Rate	Spearman’s rho	P-value	β	95% confidence interval	P-value	R^2^
PBR	0.987	<0.001	2.68	2.06 to 3.29	<0.001	0.813
EPBR	0.819	<0.001	0.05	0.03 to 0.06	<0.001	0.710
VPBR	0.643	0.002	0.07	0.03 to 0.11	0.002	0.403
MPBR	0.980	<0.001	0.29	0.23 to 0.36	<0.001	0.841
LPBR	0.991	<0.001	2.27	1.74 to 2.80	<0.001	0.809

**Figure 9:**
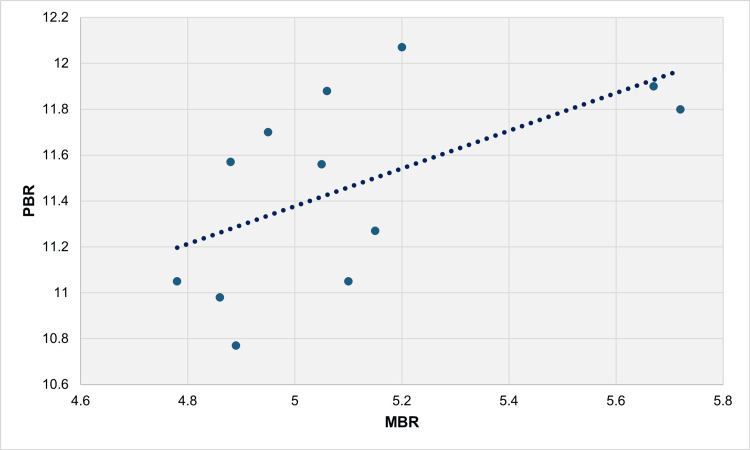
Scatter plot for PBR and MBR (per 100 live births) with the best fit regression line, 2011-2022. PBR preterm birth rate; MBR multiple birth rate

Very high correlations with MBR were observed for LPBR (rho = 0.991, p < 0.001) and MPBR (rho = 0.980, p < 0.001), while the correlation was weaker for EPBR (rho = 0.819, p < 0.001) and even more so for VPBR (rho = 0.643, p < 0.001). The R^2^ coefficients indicated that MBR variability could explain over 80% of the changes in MPBR and LPBR, but only 71.0% of EPBR and 40.3% of VPBR (Table 2). In contrast, PBR exhibited a weaker correlation with MBR in the recent period of 2011-2022, with a rho value of 0.655 (p = 0.021). The linear association was marginally statistically significant (p = 0.049), with only 33.5% of the variation in PBR being explained by the changes in MBR (Figure 9, Table 3).

**Table 3:** Correlation of preterm birth rates with the multiple birth rate in Greece, 2011-2022. PBR: preterm birth rate; EPBR: extremely preterm birth rate; VPBR: very preterm birth rate; MPBR: moderately preterm birth rate; LPBR: late preterm birth rate

Rate	Spearman’s rho	P-value	β	95% confidence interval	P-value	R^2^
PBR	0.655	0.021	0.82	0.01 to 1.64	0.049	0.335
EPBR	0.205	0.522	-0.01	-0.07 to 0.06	0.839	0.004
VPBR	-0.119	0.712	-0.04	-0.12 to 0.05	0.359	0.085
MPBR	0.923	<0.001	0.18	0.10 to 0.26	<0.001	0.730
LPBR	0.566	<0.055	0.68	-0.05 to 1.42	0.065	0.301

From 2011 to 2022, there was a very strong correlation between MPBR and MBR (rho = 0.923, p < 0.001) with an R^2^ of 0.730, while the associations of EPBR, VPBR, and LPBR with MBR were not statistically significant (Table 3).

## Discussion

In this study, very strong correlations between MBR and PBR in the Greek population for the period 1991-2022 are reported. The associations were most pronounced during the period of rapid increase in PBR in Greece (1991-2011) and for the rates of less severe prematurity (MPBR and LPBR).

The PBR in Greece saw a significant increase from 2.77 per 100 live births in 1991 to a historic peak of 12.07 per 100 live births in 2018 and subsequently settling at 11.90 per 100 live births in 2022. Between 1991 and 2011, the average annual increase was 7.3%. However, from 2011 to 2022, the rise in PBR slowed down, showing a minor, statistically non-significant upward trend of 0.5% annually [2]. Additionally, the period post-1990 witnessed a simultaneous epidemic surge in multiple pregnancies in Greece, associated with the escalating maternal age and the heightened utilization of medical-assisted reproduction services [15]. In fact, between 1990 and 2010, there was a 180% increase in the rate of live births to mothers aged 35-39 years, and the fertility rate of women aged 40-44 years surged by 2.8 times [17]. The MBR exhibited a sharp upward trend from 1991 to 2001, with an annual increase of 6.7%. This growth rate slowed down from 2001 to 2021, averaging an annual increase of 0.9% [10]. Starting at 2.27 per 100 live births in 1991, the MBR peaked at 5.72 per 100 births in 2021 before slightly decreasing to 5.67 per 100 live births in 2022.

**Figure 10:**
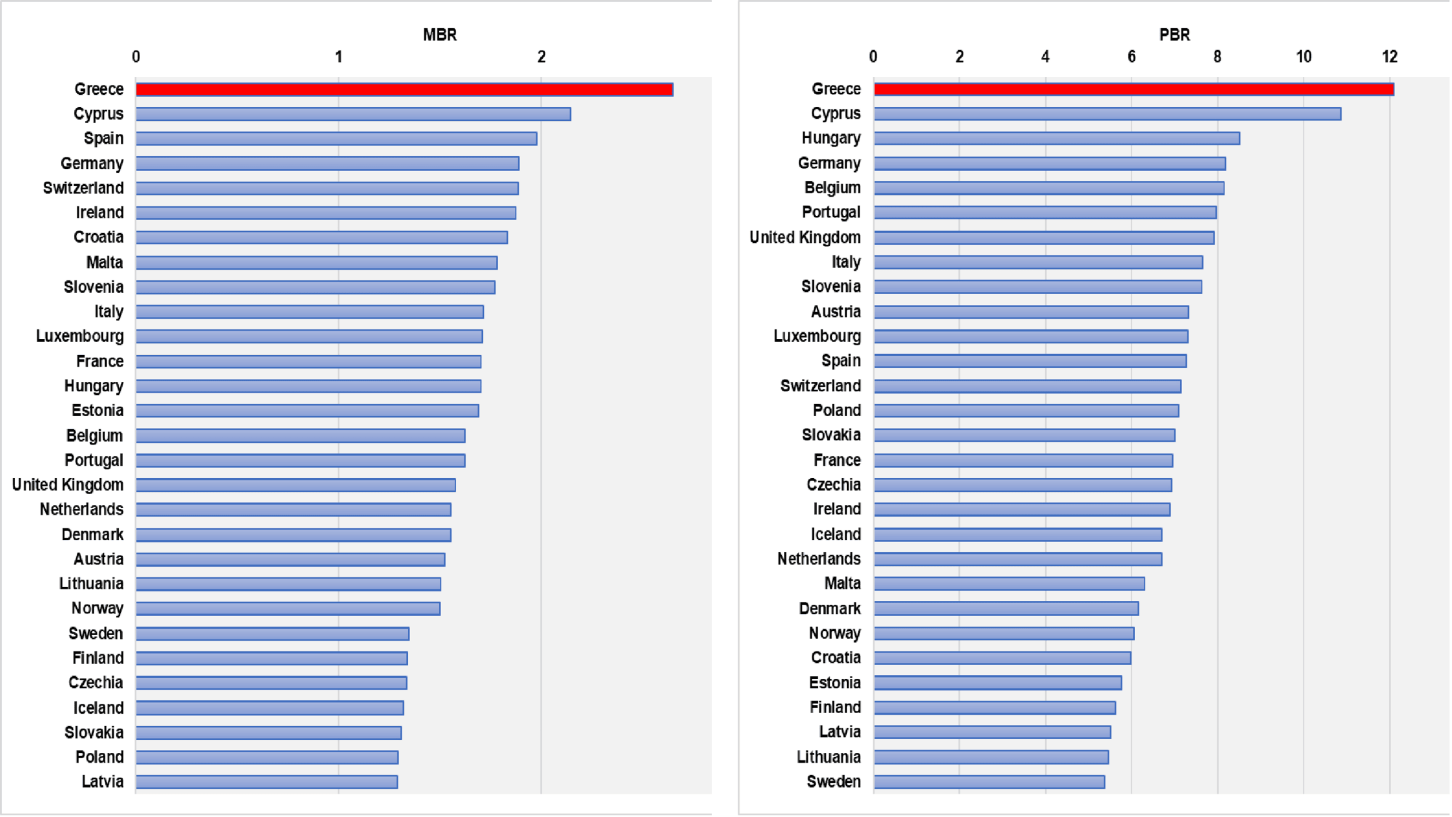
MBR and PBR data for 29 European countries for the year 2018. The MBR is expressed as per 100 maternities resulting in live birth or stillbirth. The PBR is expressed as per 100 live births. PBR: preterm birth rate; MBR: multiple birth rate

The significant increases in both PBR and MBR in Greece led to the country having the highest rates among the high-growth nations. Figure 10 illustrates Greece’s leading position in prematurity and multiplicity rates among European Union countries for the year 2018, as per data from Europeristat’s European perinatal health report [18]. Of note, data from Greece were not incorporated into this report; instead, they were included by the authors. Furthermore, the MBR of Greece was transformed to be expressed per 100 maternities resulting in live birth or stillbirth to align with the Europeristat data.

Between 1991 and 2022, a very high statistically significant positive correlation was observed between PBR and MBR in the Greek population, with Spearman’s rho value of 0.950. This correlation coefficient was notably greater than that reported in a study of 19 European countries, where the rho coefficient for the correlation of PBR with MBR for the period 1996-2008 was 0.66 [11]. Additionally, the linear regression model yielded a statistically significant β coefficient of 3.32, indicating a magnifying effect of multifetal gestations on PBR in such a manner that a one-unit increase in MBR per 100 live births led to a 3.32 increase in PBR per 100 live births. This finding may be elucidated by the gradual rise in PBR among multiple pregnancies in Greece. In fact, the multiple PBR tripled from approximately 21% to 60% between 1996 and 2008 [19]. Moreover, the R^2^ coefficient was found to be 0.838, suggesting that 83.8% of the rise in PBR can be accounted for by changes in MBR.

From 1991 to 2022, significant correlations were observed between MBR and all PBRs. The strongest correlations were found for MPBR (rho = 0.962) and LPBR (rho = 0.940), which aligns with the observation that these indicators exhibited the most pronounced increasing trends [2]. Comparatively, the correlations were more modest for EPBR and VPBR, with rho values of 0.847 and 0.810, respectively. The fluctuations in MBR explained 85.8% and 83.7% of the variations in MPBR and LPBR, while this figure was 72.5% for EPBR. The impact of multiple pregnancies on VPBR appeared to be the least significant, with an R^2^ value of 0.570.

The correlations were then analyzed separately for the period of steep PBR growth trend (1991-2011) and the plateau period (2011-2022). During 1991-2011, PBR exhibited a near-perfect correlation with MBR, with a correlation coefficient of 0.987. Similarly, LPBR and MPBR showed near-perfect correlations with MBR, with rho values of 0.991 and 0.980, respectively. The association of MBR with EPBR was also strong, with a rho of 0.819. In contrast, the weakest association of MBR was observed with VPBR, with a rho value of 0.643. In the more recent period of 2011-2022, the association of PBR with MBR remained significant but decreased to a rho value of 0.655. The linear model could only explain 33.5% of the variance in PBR during this period. This change is likely due to shifts in the use of fertility treatments, occurring against the backdrop of the deep economic recession in the country [15]. Notably, MPBR maintained a very close association with the trend in MBR during 2011-2022, with a correlation coefficient of 0.923, while the associations for the other prematurity rates were not statistically significant.

Annually, from 1992 to 2022, both PBR and MBR increased simultaneously in 17 out of 23 years. The impact of multiple pregnancies on PBR was also typically suggested by the rates observed in 2020 and 2021. In 2020, fertility treatments were limited due to strict measures imposed during the coronavirus epidemic [15]. Consequently, compared with 2019, there was a significant decrease in MBR by 0.29 per 100 live births, while PBR fell by 0.83 per 100 live births and 7%, respectively, marking the largest annual absolute and relative declines in PBR. Subsequently, in 2021, the historically largest annual absolute (0.95 per 100 live births) and relative (20%) increases in MBR were registered. This surge can be attributed to the rebound effect of catch-up infertility treatments, which also coincided with a 7% rise in PBR.

Multiple pregnancies significantly increase the risk of preterm delivery and account for a substantial proportion of preterm births in high-income countries. In the United States, in 2022, the PBR for twins was over seven times higher than that for singletons, and, overall, multiple pregnancies were responsible for about 19% of all preterm births in the country [20]. The proportion of multiple preterm births among total preterm births is likely much higher in Greece. For instance, from 2010 to 2020, in a university obstetrics department in Thessaloniki, multiple births accounted for around one-third of total preterm births [21]. A population analysis conducted across three countries from 1995 to 1997 revealed that the relative risk of preterm birth for twin compared with singleton pregnancies was 5.4 in France, 8.4 in Canada, and 9.5 in the United States, with population attributable risks (PARs) of approximately 10%, 14%, and 19%, respectively [12]. In a similar European study conducted in 2000, the PARs of plural gestations for preterm delivery varied from 18% to 25%, with multiple pregnancies carrying an 8-10 times higher risk of preterm birth [13]. A more recent analysis in 2008 revealed that the multifetal PAR for preterm birth exceeded 20% in most European countries studied, with Greece showing a particularly high PAR of over 25% [11,18]. Furthermore, another European study found that in 2010 the median relative risk of preterm birth in multiple pregnancies was nearly 10 times higher compared with single pregnancies, resulting in a total PAR of approximately 22% [22].

In economically developed nations, the increase in multiple pregnancy rates is linked to delayed motherhood. Women aged 35 and older who conceived naturally have a two- to three-fold higher chance of having multiple births, accounting for an estimated 17% of all multiple births in the United States [23,24]. However, the primary driver behind the significant rise in multiple births is the growing use of medically assisted reproduction. This phenomenon is attributed to assisted reproductive technology (ART) treatments, such as in vitro fertilization (IVF) and intracytoplasmic sperm injection, which often involve the transfer of multiple embryos. Additionally, pharmaceutical ovulation induction often leads to multiple ovulations. In 2018, it was estimated that ART was responsible for about one in eight multiple births in the United States and accounted for over 5% of all preterm births [25]. Over the four decades from the early 1970s to the late 2000s, infertility treatments contributed to a 60% rise in twin births in the United States, with the most significant increase observed in the 30-34-year age group. In 2011, medically assisted reproduction accounted for 36% of twin births, and, notably, the greatest proportion of this increase was attributed to non-ART treatments [26,27]. A study examining data from seven developed countries found that the proportion of multiple gestation births resulting from ART ranged from 15% to 30%, influenced by both the rates of single embryo transfers and the overall utilization of ART [28].

Fertility treatments, particularly ART, are anticipated to have a growing impact on MBR in Greece, which appears to be worsening. A study analyzing clinical data from 2001 to 2010 at a university obstetrics clinic in Athens found that more than 26% of twin pregnancies resulted from ART [29]. In contrast, a study examining delivery data from the past 15 years at a university obstetrics clinic in Thrace, Greece, revealed that 55% of multiple pregnancies were attributed to IVF [30]. Currently, there are no recommended evidence-based clinical interventions for secondary prevention of preterm birth in plural pregnancies [31]. Therefore, clinical practices that minimize the number of transferred embryos and promote single-embryo transfer are effective public health strategies for reducing preterm labor [32-34].

The findings of our study suggest a consistently robust positive relationship between the PBR and the MBR in Greece from 1991 to 2022. These results highlight the significant role of multiple pregnancies in the rapid increase in preterm births in the country, with more than 80% of the rise in PBR linked to the corresponding increase in MBR. Correlations with MBR were statistically significant across all preterm gestational ages, with particularly strong associations observed for the MPBR and the LPBR. The correlations were nearly perfect during the period of exponential growth in PBR (1991-2011) [35]. In contrast, during the period from 2011 to 2022, the association was significantly attenuated, suggesting that other factors had a relatively larger impact on the change in preterm births. Changes in obstetrical practices, fertility therapies, or other factors associated with the financial recession in the country may have impacted the relationship between PBR and MBR.

The strengths of this study lie in the comprehensive data derived from official nationwide birth certificate records. This enabled a detailed investigation of the relationships between PBR and MBR within the Greek population, along with a more focused analysis of the correlations between PBR and MBR based on gestational age. The associations were identified using Spearman’s rank correlation and were further validated through linear regression analysis. Conversely, a limitation of the current analysis stems from the well-documented challenges in accurately assessing gestational age, which, in turn, affects the definition of premature births [2]. Additionally, constraints arise from the ecological nature of our study, which allowed for the examination of associations at the population level rather than at the individual level. This restricted our ability to make causal inferences or consider potential confounding factors. Future research should focus on conducting more detailed and nuanced analyses to gain a deeper understanding of the effect of plural gestations on PBR trends, as well as to identify additional factors that may have contributed to the increasing trends of PBR in Greece over the past three decades.

## Conclusions

The upward trend in prematurity rates over the past three decades has shown a strong correlation with the increase in multiple births in the Greek population, suggesting a major impact of plural pregnancies on the rising trends in PBRs in the country. Future research should focus on a more detailed investigation of the effects of multiple gestations on the high rates of prematurity in Greece.
